# Genetic diversity and spatial-temporal distribution of *Yersinia pestis* in Qinghai Plateau, China

**DOI:** 10.1371/journal.pntd.0006579

**Published:** 2018-06-25

**Authors:** Xiaoqing Xu, Yujun Cui, Youquan Xin, Xiaoyan Yang, Qingwen Zhang, Yong Jin, Haihong Zhao, Jian He, Xing Jin, Cunxiang Li, Juan Jin, Xiang Li, Haisheng Wu, Zhizhen Qi

**Affiliations:** 1 Qinghai Institute for Endemic Disease Prevention and Control, Xining, People's Republic of China; 2 Key Laboratory for Plague Prevention and Control of Qinghai Province, Xining, People's Republic of China; 3 State Key Laboratory of Pathogen and Biosecurity, Beijing Institute of Microbiology and Epidemiology, Beijing, People's Republic of China; Sichuan University, CHINA

## Abstract

**Background:**

Plague, caused by the bacterium *Yersinia pestis*, is a highly infectious, zoonotic disease. Hundreds of human plague cases are reported across the world annually. Qinghai Plateau is one of the most severely affected plague regions in China, with more than 240 fatal cases of *Y*. *pestis* in the last 60 years. Conventional epidemiologic analysis has effectively guided the prevention and control of local plague transmission; however, molecular genetic analysis is more effective for investigating population diversity and transmission. In this report, we employed different genetic markers to analyze the population structure of *Y*. *pestis* in Qinghai Plateau.

**Methodology/Principal finding:**

We employed a two-step hierarchical strategy to analyze the phylogeny of 102 Qinghai Plateau isolates of *Y*. *pestis*, collected between 1954 and 2011. First, we defined the genealogy of *Y*. *pestis* by constructed minimum spanning tree based on 25 key SNPs. Seven groups were identified, with group 1.IN2 being identified as the dominant population. Second, two methods, MLVA and CRISPR, were applied to examine the phylogenetic detail of group 1.IN2, which was further divided into three subgroups. Subgroups of 1.IN2 revealed a clear geographic cluster, possibly associated with interaction between bacteriophage and *Y*. *pestis*. More recently, *Y*. *pestis* populations appear to have shifted from the east toward the center and west of Qinghai Plateau. This shift could be related to destruction of the local niche of the original plague focus through human activities. Additionally, we found that the abundance and relative proportion of 1.IN2 subgroups varied by decade and might be responsible for the fluctuations of plague epidemics in Qinghai Plateau.

**Conclusion/Significance:**

Molecular genotyping methods provided us with detailed information on population diversity and the spatial-temporal distribution of dominant populations of *Y*. *pestis*, which will facilitate future surveillance, prevention, and control of plague in Qinghai Plateau.

## Author summary

Plague is a highly infectious disease caused by the *Yersinia pestis* bacterium. Since the first strain of *Y*. *pestis* was isolated in Qinghai in 1954, confirmed plague cases have occurred nearly every year, and more than 240 people have died from plague over the past 60 years. In this study, we analyzed 102 *Y*. *pestis* strains collected from Qinghai Plateau between 1954 and 2011. We determined their genetic diversity and inferred their spatial-temporal distribution, based on genetic markers including SNPs, VNTRs and CRISPRs. Our results indicate that 1.IN2 is the dominant group of *Y*. *pestis* in Qinghai Plateau, and its three subpopulations revealed clear geographic clustering that might be driven by interaction with bacteriophages. We observed that the *Y*. *pestis* population has moved from the east of Qinghai Plateau to central and western regions over the past 60 years. We also found that the abundance and relative proportion of 1.IN2 subgroups varied over time, leading to fluctuations in plague epidemics. These results extend our knowledge of the genetic diversity of *Y*. *pestis*, and its population dynamics in natural plague foci over a number of years. With ongoing risk of outbreaks, we recommend enhanced surveillance in this region.

## Introduction

Plague, caused by the virulent bacteria *Yersinia pestis*, is a highly infectious zoonotic disease [1, 2]. Human infection is usually caused by direct contact with infected animals or fleas and is fatal without prompt antibiotic treatment. Three major plague pandemics have been documented in history, which not only led to millions of deaths but also facilitated worldwide spread of *Y*. *pestis*, causing virtual global colonization, except for in Australia and Antarctica [3]. Currently, *Y*. *pestis* circulates between multiple species of rodent hosts and species of flea vectors, and persists in multiple natural plague foci in Asia, Europe, Africa, and America, causing hundreds of human plague cases annually.

Qinghai Plateau is one of the most severely affected plague regions in China and over 200, 000 km^2^ of this territory is covered by natural plague foci. It is reported that at least 20 species of mammals and 11 species of fleas could be infected by *Y*. *pestis* in Qinghai Plateau [4]. Some of them, such as *Marmota himalayana*, act as a reservoir, maintaining *Y*. *pestis* transmission in the environment. The first isolate of *Y*. *pestis* in Qinghai Plateau was from *M*. *himalayana* in 1954 in Guide County, after the establishing of a routine surveillance system in Qinghai Plateau in the same year. Since then, two types of natural plague foci, characterized by different main hosts, *Marmota himalayana* and *Microtus fuscus*, have been identified.

Human plague cases have been reported every year from 1954 to 2014 except for 1972, 1984, 1999, 2000, 2002, 2007, 2008, 2010, 2012, 2013, and 2014 in Qinghai Plateau, and more than 240 people have died from plague during this period [4]. In the early years since surveillance was established, human cases in Qinghai Plateau were associated with marmot hunting, which was an important means of livelihood for many local communities. In recent years, numbers of human cases have declined following the official prohibition of marmot hunting. Instead, a new disease pattern amongst marmots, livestock and humans has challenged our understanding of plague transmission. For example, an outbreak of primary human pneumonic plague in 2009 in Xinghai County was introduced by an infected dog [5]. Therefore, the threat of human plague remains, and effective control measures are still required.

Previously, researchers have proposed many methods for local plague control, informed by conventional plague epidemiology [6, 7]. However, it is difficult to trace the source of isolates precisely, to uncover the transmission dynamics of the isolates and to analyze the population structure and epidemiological characteristics, because of the difficulty in typing of *Y*. *pestis*, which is generally regarded as lacking much genetic variation within the species [8].

Molecular genotyping and phylogenetic analysis are useful analytic methods that are highly effective at increasing understanding of genetic relationships and molecular epidemiology. Multiple molecular methods have been applied to genotyping *Y*. *pestis*, including SNP (Single Nucleotide Polymorphism) [9–11], MLVA (Multiple Locus VNTR Analysis) [12–14], CRISPR (Clustered Regularly Interspaced Short Palindromic Repeat) [15, 16], DFR (Different Region Analysis) [17], and IS (Insertion Sequence) [18]. These methods each have their own advantages and disadvantages for phylogenetic analysis. For example, data from genome-wide SNPs provide the highest resolution, but the cost of genome sequencing numerous samples from a population remains high. Use of a small subset of SNPs, as in our study, provides relatively low resolution. The same applies to the CRISPR method, because only three spacer arrays are available in *Y*. *pestis* [19, 20]. MLVA seems to provide a high resolving power, but the high mutation rate of VNTR (Variable Number Tandem Repeat) loci leads to a high homoplasy rate in phylogeny, reducing the reliability of deep branches [21]. The method that combines both the SNP and MLVA markers largely avoids the limitations associated with each method and has been successfully applied to plague epidemiologic analyses in Madagascar, providing reliable and high resolution phylogeny [20, 22].

In this study, we introduced a hierarchical strategy based on SNP, MLVA and CRISPR methods, to investigate the population diversity of *Y*. *pestis* in Qinghai Plateau, and to correlate the geographic distribution with different lineages of this pathogen.

## Methods

### Bacteria and DNA extraction

*Y*. *pestis* strains were collected from 32 counties in Qinghai Plateau, between 1954 and 2011, during routine plague surveillance. For each county, if fewer than five strains were isolated since the initiation of surveillance, all historical isolates were used. For the county with more available *Y*. *pestis* isolates, five or six strains from different host/vector and sampling periods were selected. In total, 102 strains were used in this study (Table 1).

**Table 1:** Overview of *Y*. *pestis* isolates in Qinghai Plateau.

Strain ID	Phylo-group	Year of isolation	Source of isolates	County
00056	1.IN2	1965	Patient	Huangzhong
00125	1.IN2	1967	*Marmota himalayana*	Gonghe
00130	1.IN2	1967	*Callopsylla dolabris*	Menyuan
00282	1.IN2	1970	*Marmota himalayana*	Dulan
00352	1.IN2	1974	*Marmota himalayana*	Gangca
00497	1.IN2	1980	*Marmota himalayana*	Qumarleb
00559	1.IN1	1982	Human body	Madoi
00564	1.IN2	1982	Human body	Menyuan
00578	1.IN1	1983	Human body	Madoi
00609	1.IN2	1986	*Marmota himalayana*	Tongren
00626	1.IN2	1986	*Callopsylla dolabris*	Tongren
00718	1.IN2	1993	Human body	Chindu
01058	1.IN2	1960	*Vulpes*	Gonghe
01069	1.IN2	1964	Patient	Gonghe
01094	1.IN2	1971	*Marmota himalayana*	Gonghe
02010	1.IN2	1958	Human body	Qilian
02040	1.IN2	1965	*Marmota himalayana*	Qilian
02054	1.IN2	1980	*Marmota himalayana*	Qilian
02062	1.IN2	1987	*Marmota himalayana*	Qilian
02064	0.PE4	2004	Human body	Qilian
02067	1.IN1	2011	Human body	Qilian
03001	1.IN2	1954	*Ochotonidae curzoniae*	Henan
04003	1.IN2	1959	Patient	Gangca
04008	1.IN2	1960	*Marmota himalayana*	Gangca
04019	1.IN2	1973	Patient	Gangca
05010	0.PE7	1962	Human body	Xinghai
05015	1.IN2	2009	Patient	Xinghai
05020	1.IN2	2009	Canine	Xinghai
06002	1.IN2	1976	Human body	Tongren
06003	1.IN2	1960	Human body	Tongde
07001	1.IN1	1957	*Marmota himalayana*	Jainca
08008	1.IN2	1971	*Marmota himalayana*	Tianjun
08014	1.IN2	1979	*Marmota himalayana*	Tianjun
08017	1.IN2	2003	Human body	Tianjun
08021	1.IN2	2008	*Marmota himalayana*	Tianjun
09001	1.IN1	1979	*Marmota himalayana*	Mang'ai
10001	1.IN2	1957	*Marmota himalayana*	Haiyan
10012	1.IN2	1964	*Dermacentor nuttalli*	Haiyan
10023	1.IN2	1968	Human body	Haiyan
10028	1.IN2	1983	*Marmota himalayana*	Haiyan
11001	1.IN1	1954	*Marmota himalayana*	Guide
11003	1.IN2	1985	*Marmota himalayana*	Guinan
11004	1.IN2	1985	*Dermacentor nuttalli*	Guinan
12003	2.MED3	1961	*Marmota himalayana*	Menyuan
12008	1.IN2	1964	*Marmota himalayana*	Menyuan
13001	0.PE4	1961	Human body	Dulan
13014	1.IN2	1980	*Marmota himalayana*	Dulan
13016	1.IN1	1994	*Marmota himalayana*	Dulan
14004	1.IN2	1971	Human body	Huangyuan
14005	1.IN2	1970	*Marmota himalayana*	Huangyuan
15001	1.IN2	1963	Human body	Yushu
15017	1.IN2	1975	*Ovis aries*	Yushu
15041	1.IN2	1986	*Marmota himalayana*	Yushu
15067	1.IN2	2007	*Vulpes*	Yushu
16001	1.IN2	1964	Human body	Zadoi
16005	1.IN2	1974	Human body	Zadoi
16012	1.IN2	1988	Human body	Zadoi
16013	1.IN2	1989	Human body	Zadoi
17001	1.IN2	1964	Human body	Zhidoi
17003	1.IN2	1978	Canine	Zhidoi
17009	1.IN2	2003	*Ovis aries*	Zhidoi
17019	1.IN2	2007	*Marmota himalayana*	Zhidoi
18001	1.IN2	1980	*Marmota himalayana*	Chindu
18019	0.PE4	2001	*Microtus fuscus*	Chindu
18023	0.PE4	2004	Patient	Chindu
19014	1.IN2	1976	*Marmota himalayana*	Wulan
19023	1.IN2	1979	*Marmota himalayana*	Wulan
19030	1.IN2	1996	*Marmota himalayana*	Delhi
19036	1.IN2	1995	Patient	Delhi
19058	1.IN2	2000	*Rhadinopsylla liventricasa*	Delhi
19085	1.IN2	2002	*Marmota himalayana*	Delhi
19099	3.ANT1	2004	Patient	Wulan
19100	1.IN2	2004	Human body	Wulan
19121	1.IN2	2011	*Marmota himalayana*	Delhi
20005	1.IN2	1968	*Marmota himalayana*	Tanggula
20009	1.IN2	1978	Human body	Tanggula
20014	1.IN2	1989	Human body	Tanggula
20020	1.IN2	1999	*Marmota himalayana*	Tanggula
20045	1.IN2	2007	*Marmota himalayana*	Tanggula
21002	1.IN2	1970	*Marmota himalayana*	Xunhua
22002	1.IN2	1970	*Marmota himalayana*	Guide
22004	1.IN2	1965	Patient	Guide
23005	1.IN2	1980	Human body	Qumarleb
23006	1.IN2	1986	Human body	Qumarleb
23009	1.IN2	2005	Human body	Qumarleb
24002	1.IN1	1978	*Marmota himalayana*	Madoi
25001	1.IN2	1978	*Marmota himalayana*	Zêkog
25010	1.IN2	1991	*Allactaga sibirica*	Zêkog
25012	1.IN2	1991	*Marmota himalayana*	Zêkog
26001	3.ANT1	1972	*Oropsylla silantiewi*	Lenghu
26005	3.ANT1	1972	*Marmota himalayana*	Lenghu
27001	1.IN2	1958	Human body	Datong
27002	1.IN2	1991	*Marmota himalayana*	Tongde
27004	1.IN2	2001	*Vulpes*	Tongde
27005	1.IN2	2001	Human body	Tongde
28023	1.IN2	1986	*Marmota himalayana*	Nangqên
28025	1.IN2	1990	*Marmota himalayana*	Nangqên
28032	1.IN2	1997	*Ovis aries*	Nangqên
28036	2.ANT2	2004	Human body	Nangqên
29001	1.IN2	1978	*Marmota himalayana*	Maqên
29002	1.IN2	1979	*Lxodoidea*	Maqên
29003	1.IN2	1980	Human body	Maqên

Bacteria strain and its background information used in this study were provided by the Bacteria Specialized Laboratory of *Yersinia pestis*, Medical Bacteria Center of Management and Preservation, China. The *Y*. *pestis* cultures were incubated in Luria-Bertani medium at 28°C for 48 hours. Genomic DNA was then extracted using the conventional SDS-lysis and phenol-chloroform method.

### SNP genotyping

Overall, 25 SNP loci (S1 Fig and S1 Table) were selected to genotype the isolates of *Y*. *pestis* from Qinghai Plateau. An economical and timesaving PCR method, using the GenoType *Tsp* DNA Polymerase, was developed to identify the nucleotide status of SNP loci in this study. Brief details of the principle and procedure of the PCR are shown in S2 Fig, and the primers are listed in S1 Table.

PCR was performed in a mixture of 15 μl volumes containing 100 ng of DNA, 0.5 μM of each primer, 0.2 mM dNTPs, 1.5 mM MgCl_2_ and 1 U *Tsp* DNA polymerase (Cat. *No*.: 11448–032, Invitrogen, USA). Amplification took place under the following conditions: pre-denaturation at 94°C for 1 min 30 s; then 10 cycles of 30 s at 94°C, 30 s at 50°C and 1 min at 72°C; 20 cycles of 30 s at 89°C, 30 s at 50°C and 1 min at 72°C; finally, an extension at 72°C for 10 min. The products underwent electrophoresis by agarose gel at 100 V for 20 min and the visualized result recorded as “0” (negative) or “1” (positive), respectively.

Additionally, conventional PCR and DNA sequencing using the Sanger method were employed to verify the SNPs identified. All the primers (S2 Table) were designed with reference to the CO92 strain genome. PCR was performed using the recommended PCR mixture and conditions by using the *TaKaRa Ex Taq* DNA polymerase (Code *No*.: RR001, TaKaRa). The purified PCR products were then sequenced with the Applied Bio-systems 3730 automated DNA Sequencer. SNPs were identified by comparison with the allelic genes of *Y*. *pseudotuberculosis* strain IP32953, which is regarded as the most recent common ancestor (MRCA) of *Y*. *pestis*, using the DNAstar software package (DNAstar Inc., Madison, WI, USA).

### Determining the diversity of selected tandem repeat loci

A total of 19 VNTR loci (S3 Table) were selected to screen the diversity of *Y*. *pestis* isolates by the capillary electrophoresis method, using an ABI 370 sequencer, as described previously [13, 14]. The PCR products were labeled with four different fluorescent dyes (Rox, 6-Fam, Hex, and Tamra). Amplicon sizes were monitored and calculated using Genemapper 4.0 software (Applied Biosystems, Foster City, CA, USA).

The strain CO92 (GenBank accession number: AL590842) was used as a reference to estimate the motif copy number of VNTR loci for each isolate. The copy number was calculated using the following formula: *R = Rc+(L–Lc)/U*, where *R* is the motif copy number of test isolates of *Y*. *pestis*, *Rc* is the copy number in the allele of the strain CO92, *L* is the allele length (bp) of test isolates, *Lc* is the allele length (bp) of the strain CO92, and *U* is the base number of the motif.

### CRISPR analyses

Three CRISPR loci (YPa, YPb, and YPc) of 102 *Y*. *pestis* isolates were amplified using primers that separately targeted their flanking regions as described by Cui *et al* [16]. The PCR products were sequenced by using the Sanger method, and sequence assembly was performed using the Seqman module in the DNAstar package. The spacer identification and analysis of each CRISPR locus sequence was performed using the online tool CRISPRfinder (http://crispr.i2bc.paris-saclay.fr/), referring to the most recently published CRISPR spacer dictionary [22]. The nomenclature and abbreviation of CRISPR spacers were as described previously [16].

### Phylogenetic analyses

Phylogenetic analyses introduced a two-step hierarchical strategy to explore the genetic diversity and population structure of *Y*. *pestis* in Qinghai Plateau. First, we constructed the minimum spanning tree (MSTree), based on binary character data of 25 SNPs of 102 *Y*. *pestis* isolates. Second, for the dominant SNP-defined group 1.IN2, the MLVA cluster analyses were performed using the Ward method and a CRISPR dendrogram, rooted as the basal composition (a1-a2-a3-a4-a5-a6-a7, b1-b2-b3-b4 and c1-c2-c3) of CRISPR loci, was created manually. The clustering procedure based on CRISPR spacers was performed according to the hypothesis that these spacers were originally from bacterial phage that carried their homologous sequences, i.e. the bacteria strains that carried the same spacer array would have same exposure history to different lineages of phages [16]. Both the MSTree and MLVA dendrogram were built using the software BioNumerics 6.6 (Applied Maths, Belgium). The geographic distributions of strains were mapped using ArcGIS 10.2 (ESRI, Redlands, CA, USA).

## Results

### Genotyping of Qinghai Plateau isolates based on SNPs

**Fig 1:**
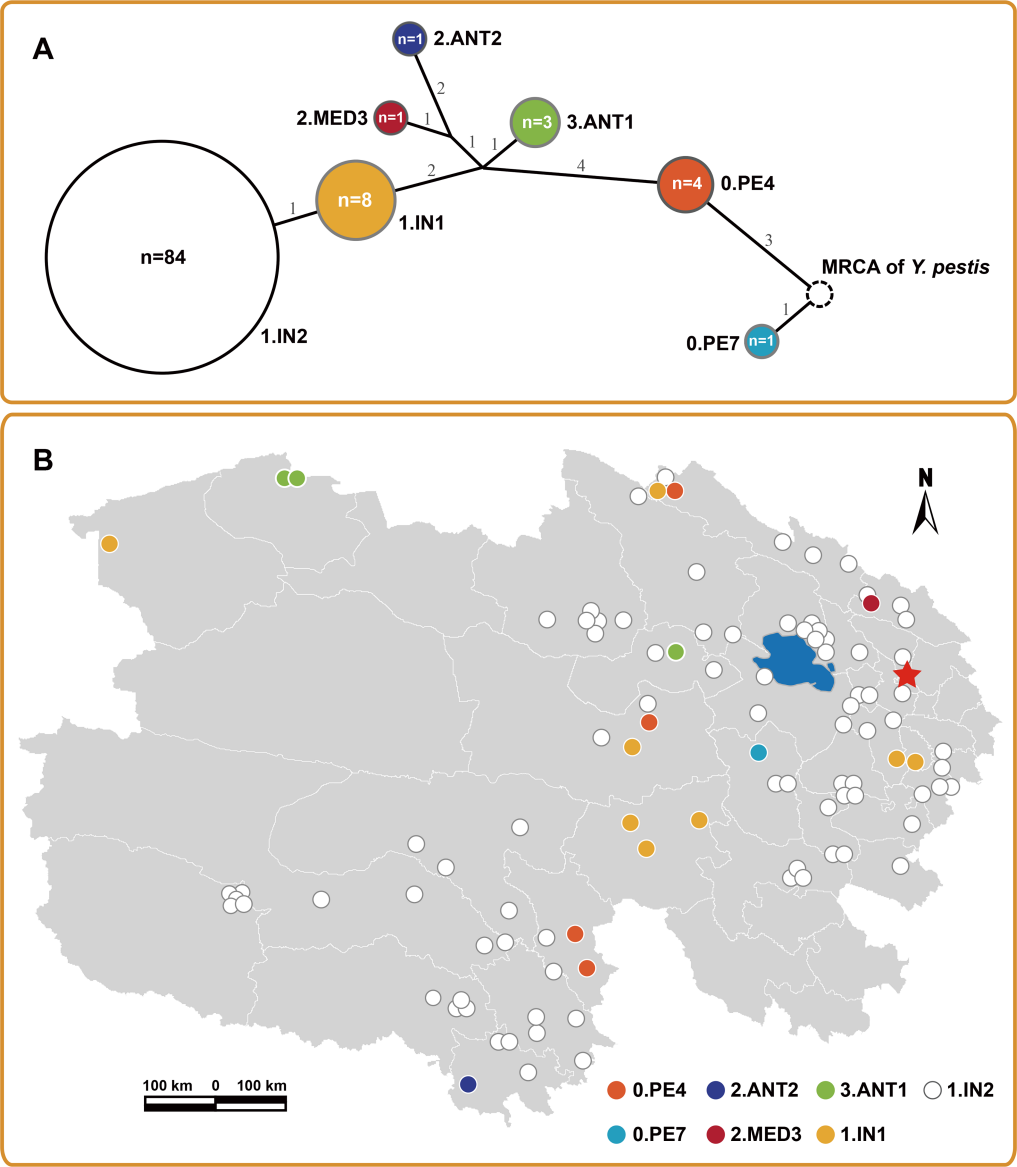
Phylogeny based on SNPs and the geographic distribution of 102 isolates of *Y*. *pestis* in Qinghai Plateau. (A) Minimum spanning tree (MSTree) based on 25 SNPs. The dotted-line circle represents the most recent common ancestor (MRCA) of *Y*. *pestis*. Colored circles indicate the non-dominant groups of *Y*. *pestis* and the white circle indicates the dominant group identified in Qinghai Plateau. The numbers on the branches indicate the SNP distance between nodes. It is important to note that the SNP distance in the MSTree does not represent the actual genetic distance between groups, because only a limited number of SNP loci were selected to be used in the analysis. The number of isolates in each group is indicated within each circle. (B) Geographic distribution of SNP lineages in Qinghai Plateau. Colors of circles correspond to the colors of SNP groups in the MSTree. The five-pointed star marks the capital city of Qinghai province. All of the maps used this paper were created with ArcGIS software based on the public geographical data downloaded from OpenStreetMap (http://www.openstreetmap.org/).

In this study, we employed key SNPs selected from previous research [9, 10] to screen 102 isolates from Qinghai Plateau, to understand the phylogenetic structure of *Y*. *pestis* in this region. Seven groups (0.PE7, 0.PE4, 2.MED3, 2.ANT2, 3.ANT1, 1.IN1, and 1.IN2) were recognized according to the MSTree based on 25 SNPs (Fig 1A). Of these isolates, 84 were attributed to 1.IN2, representing the dominant population (~82.3%) of *Y*. *pestis* in Qinghai Plateau. We also identified eight isolates belonging to the group 1.IN1, which differed from group 1.IN2 in terms of the ancestral state of the SNP s1201 (S1 Fig). The remaining 10 isolates were independently attributed to the other five groups, with four and three strains in 0.PE4 and 3.ANT1, respectively, and one isolate in each of the other three groups (Fig 1A and S4 Table).

Analysis of the geographic distribution of isolates showed that the majority were from central-eastern and southern regions of Qinghai Plateau, with few isolated from north-western parts of Qinghai Plateau and the Tanggula region (Fig 1B and S3 Fig). Qinghai Plateau is surrounded by multiple natural plague foci (S4 Fig) [17], including a *Marmota himalayana* plague focus in the Gangdisi Mountains (Focus G), a *Spermophilus dauricus alaschanicus* plague focus of the Loess Plateau in Gansu and Ningxia provinces (Focus J), a *Marmota himalayana* plague focus in the Kunlun Mountains (Focus K), and a *Microtus fuscus* plague focus in Qinghai and Sichuan provinces (Focus M). Strains from groups including 2.ANT2, 2.MED3, 2.MED2, 3.ANT1, and 0.PE4 have frequently been isolated in these plague foci [9, 10, 13, 14, 16, 17]. Therefore, the majority of the non-dominant populations identified, including 0.PE4, 2.ANT2, 2.MED3, and 3.ANT1, may have been introduced by trans-regional diffusion events between adjacent plague foci. Considering previous observations [10], ten 1.IN1 strains have been identified to date, of which nine have been isolated from Qinghai Plateau and only one from Xinjiang Province, suggesting that Qinghai Plateau is the main focus of group 1.IN1 strains, with export to other regions only occurring occasionally.

Of all 102 strains from Qinghai Plateau, only one was identified as belonging to group 0.PE7, the oldest extant lineage of *Y*. *pestis*. Until now, only three 0.PE7 strains have been identified, and all were from Xinghai County in Qinghai Plateau, identified during the 1960s [10]. This limited number of identified 0.PE7 strains suggests a very small population size, or even extinction of this ancient lineage of *Y*. *pestis* strains.

### Population diversity and phylogeography of the dominant group 1.IN2 based on MLVA and CRISPR analysis

**Fig 2:**
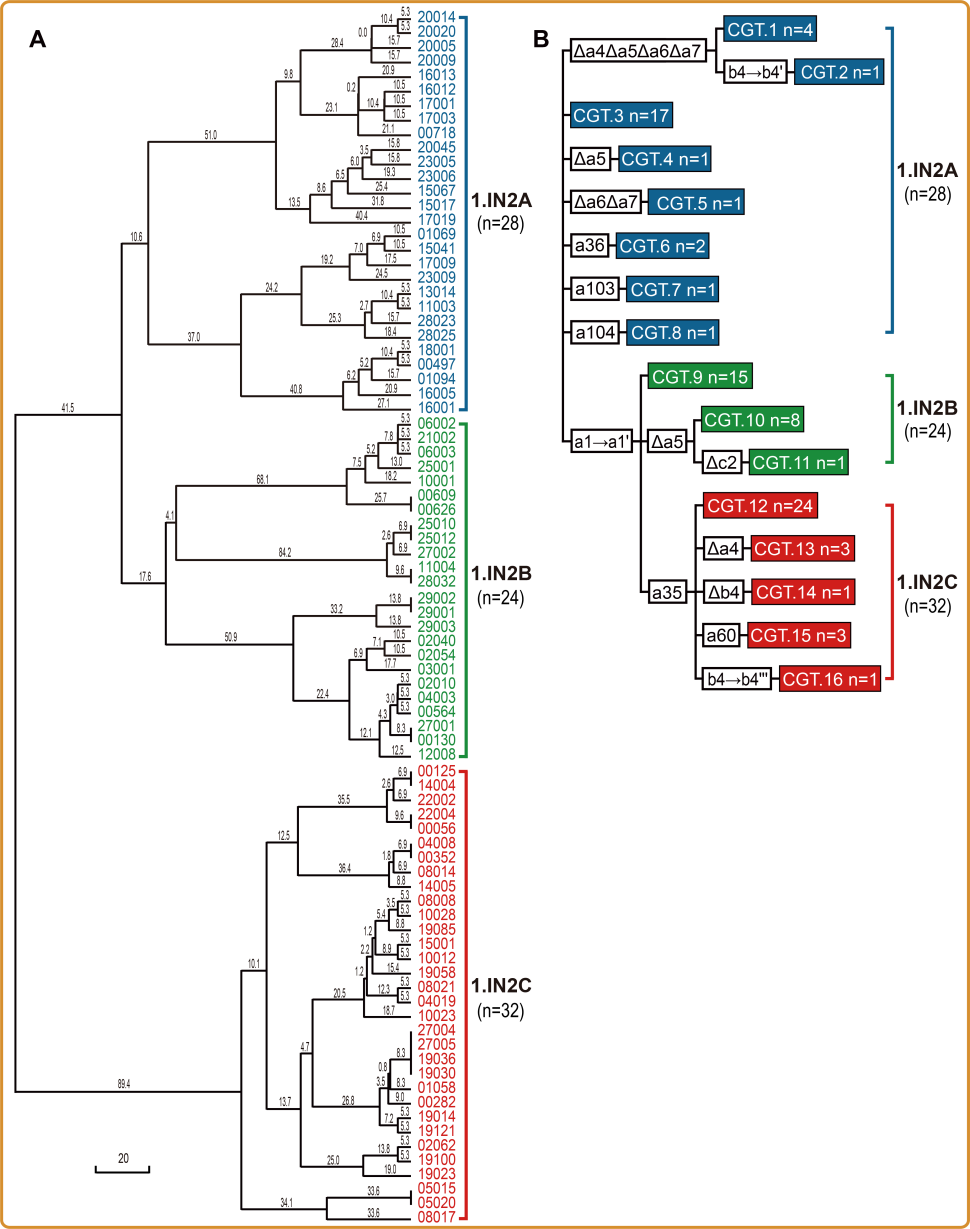
Dendrograms of 1.IN2 strains based on MLVA and CRISPR results. (A) The cladogram was generated using the Ward method [14], based on the 19 VNTR loci. Three subgroups were identified and labeled with different colors. Numbers on branches indicate the genetic distance calculated using the Ward method. (B) Dendrogram of CRISPR results (drawn manually). A conserved spacer array (a1-a2-a3-a4-a5-a6-a7, b1-b2-b3-b4 and c1-c2-c3) was assigned as the basal composition of CRISPR loci for 84 strains in this study. The triangles preceding a spacer indicate that the spacer is absent from the basal composition in the strains of corresponding lineage. Arrow lines between two spacers indicate that the latter spacer replaced the former.

To investigate population diversity amongst isolates of the dominant population, we applied MLVA and CRISPR methods to screen isolates from group 1.IN2. Based on the diversity of 19 VNTR loci, all eighty-four 1.IN2 strains were clustered into three subgroups, named 1.IN2A, 1.IN2B and 1.IN2C, and 72 genotypes were identified (Fig 2A). The CRISPR analysis revealed lower resolution than MLVA, with only 16 genotypes identified, but the subgroup clustering results were largely consistent with MLVA (Fig 2B and S4 Table). We also found that specific spacer composition, with a35 for 1.IN2C and a1’ but not a35 for 1.IN2B, can be used to distinguish the subgroups.

**Fig 3:**
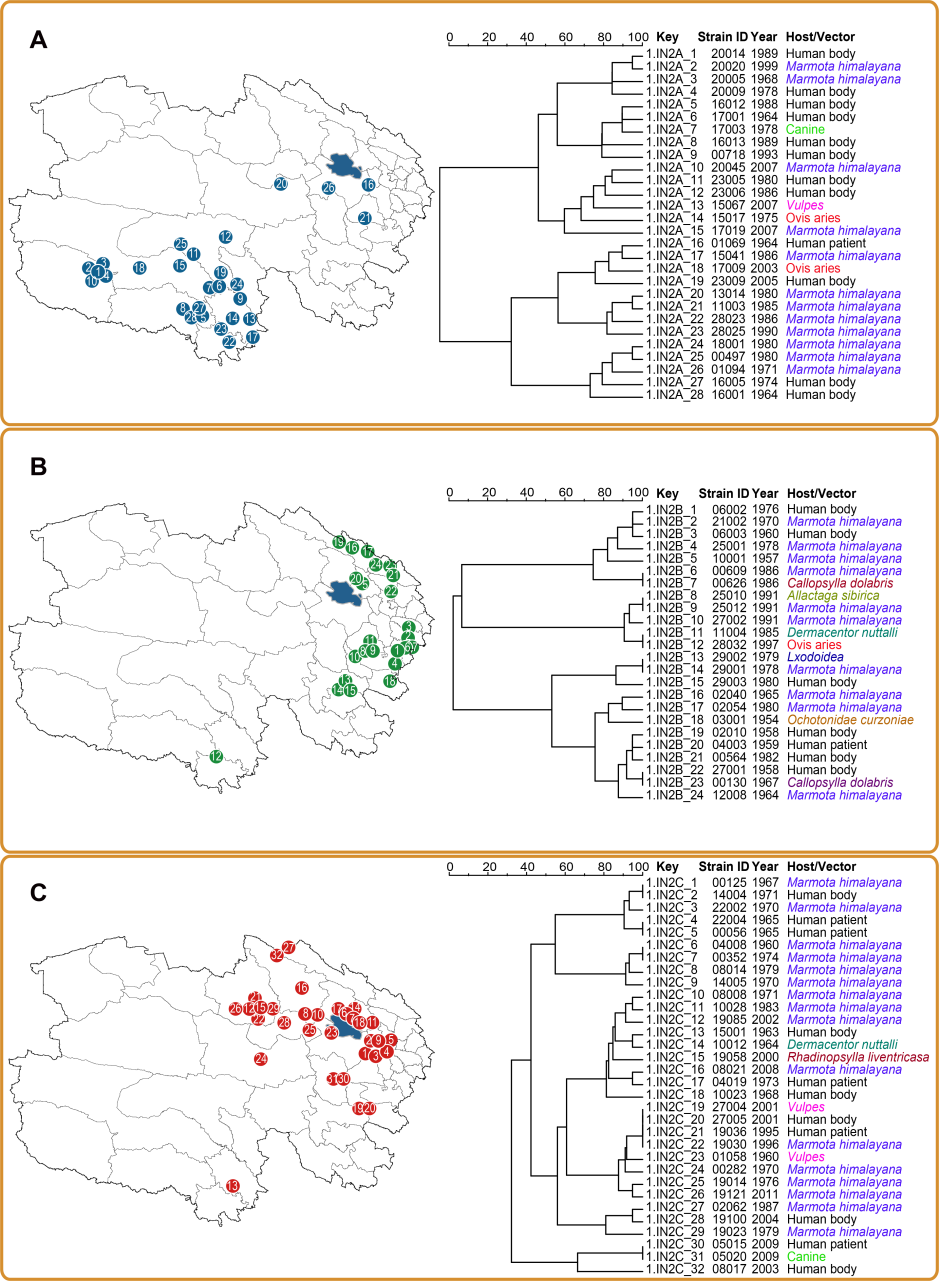
Phylogeographic analysis of group 1.IN2 *Y*. *pestis* in Qinghai Plateau. The geographical distribution and phylogeny of subgroups 1.IN2A (A), 1.IN2B (B), and 1.IN2C (C) are shown. The phylogenetic trees were built based on the diversity of 19 VNTRs. Each strain on the phylogenetic tree (see column “Key”) is numbered and correspondingly plotted on the map of Qinghai Plateau. Colors in the right column highlight different hosts or vectors of *Y*. *pestis*.

All three 1.IN2 subgroups showed a clear geographic clustering pattern, with only a few strains isolated far from the location of their major population (Fig 3). The majority of 1.IN2A strains (19 of 28, 67.9%, Fig 3A) were isolated in the Yushu Plateau (Region A in S3 Fig), with five of the remaining strains isolated at Tanggula region, the west plateau to the Yushu Plateau, and the other four strains at the southern part of Qinghai Lake (Fig 3A). Four out of five of the 1.IN2A strains (1.IN2A_1–4 in Fig 3A), located in the southwest of Qinghai Plateau, formed a monophyletic cluster in the cladogram of MLVA. Concerning the isolation time of the four strains, this cluster of strains may have been sustained in the same locality for over 30 years. However, the four strains located in the region surrounding Qinghai Lake and the Huangnan region (1.IN2A_16, 1.IN2A_20, 1.IN2A_21 and 1.IN2A_26 in Fig 3A), scattered at different branches on the cladogram of MLVA, suggesting that these four strains were very likely to have spread from Yushu Plateau to the isolation location through independent events. Interestingly, three of four strains were isolated from *M*. *himalayana*, implying the role this species may have in long distance transmission of *Y*. *pestis*. Subgroup 1.IN2B (Fig 3B) was distributed in two separate regions, one located at the southern foot of the Qilian Mountains (Region C in S3 Fig) and the second located in Huangnan region (Region D in S3 Fig). Notably, one 1.IN2B strain (1.IN2B_12 in Fig 3B), which was isolated from *Ovis aries* (Tibetan sheep), was located at the southern edge of Qinghai Plateau, which is a long distance from the other 1.IN2B populations. As domestic livestock, Tibetan sheep have a very close relationship with humans, implying that the long distance spread of this strain may be related to human activity. Subgroup 1.IN2C (Fig 3C) was mainly distributed encircling Qinghai Lake and expanded to both the west and east sides (Region B in S3 Fig). Only one 1.IN2C strain (1.IN2C_13 in Fig 3C), sourced from humans was isolated at the most southern part of Qinghai Plateau.

### Spatial-temporal distribution of *Y*. *pestis* 1.IN2 subgroups in Qinghai Plateau

**Fig 4:**
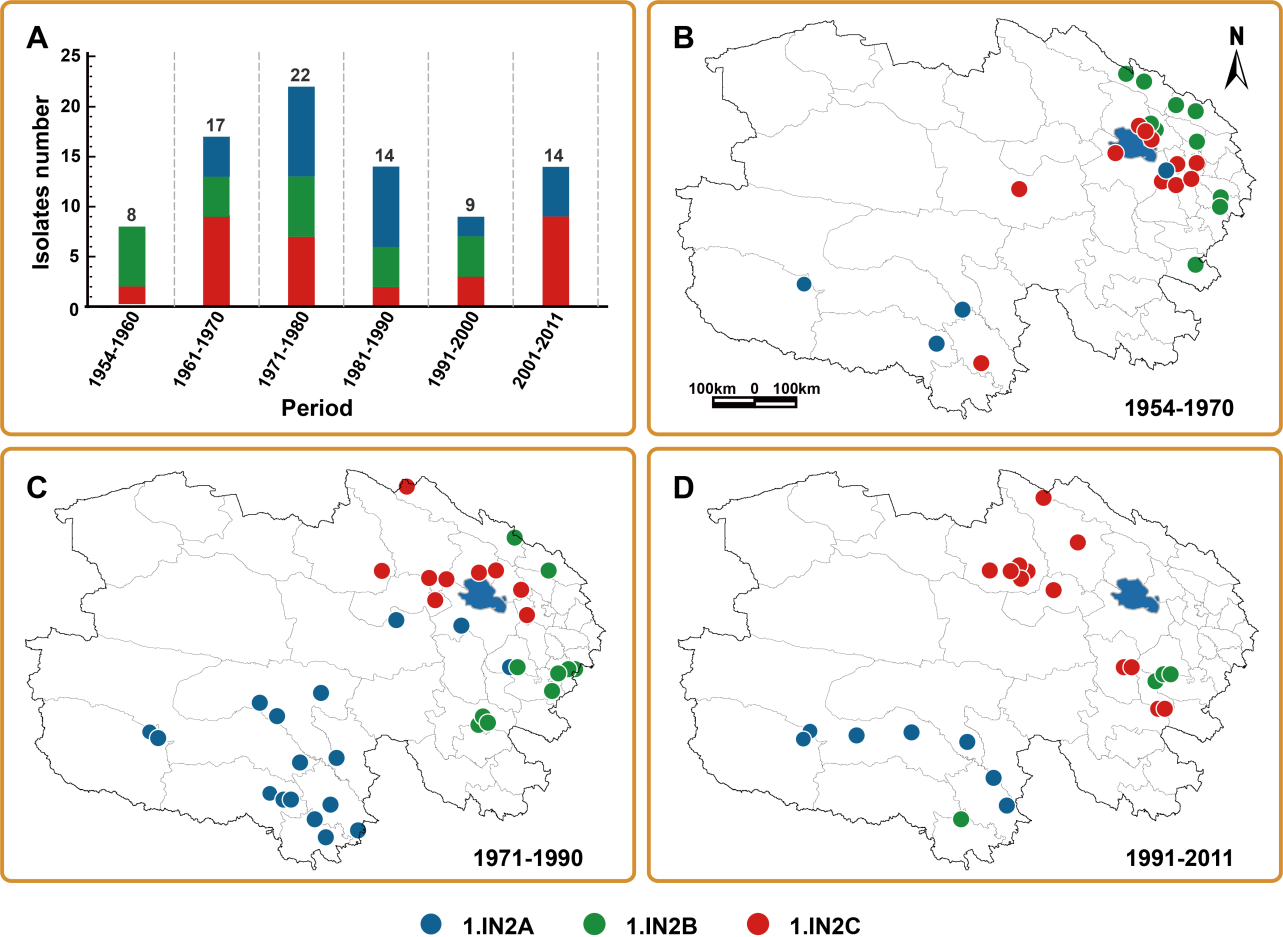
Spatial-temporal distribution of 1.IN2 isolates in Qinghai Plateau, over time. (A) Number of *Y*. *pestis* 1.IN2 group strains isolated during different time periods. Remaining panels show the geographical distributions of subgroups of 1.IN2 strains during (B) 1954–1970, (C) 1971–1990, and (D) 1991–2011. The colors reflect the different subgroups of 1.IN2 and correspond to MLVA- and CRISPR-identified clusters.

As the dominant group of *Y*. *pestis*, the number of 1.IN2 strains isolated each year can be used as an indicator of plague prevalence in Qinghai Plateau. Accordingly, phylogeographic analysis of 1.IN2 subgroups isolated during different time periods was conducted to explore the epidemiology of plague over the past 60 years in Qinghai Plateau (Fig 4). Evaluating trends in prevalence of the entire group of 1.IN2 strains between 1954 and 2011 demonstrates the periodic plague outbreaks that have occurred in Qinghai Plateau in this time (Fig 4A). The frequency of plague outbreaks increased between 1954 and 1980, reaching its peak in the 1970s. Plague prevalence then declined during the 1980s and 1990s, with plague cases increasing again after 2001.

Our molecular epidemiologic analysis has also revealed an interesting dynamic fluctuation in prevalence of each subgroup of 1.IN2 in different periods (Fig 4B–4D). Only a few 1.IN2A strains were isolated before 1970 (Fig 4B). During 1971–1990, this subgroup was frequently isolated in Yushu Plateau and several strains were identified at locations far from its major population. After the 1990s, the population of 1.IN2A strains appears to have decreased substantially and was only isolated in Yushu Plateau. Both 1.IN2B and 1.IN2C subgroups seem to have shifted to new locations over recent decades. Before 1970, most 1.IN2B strains were located at the southern foot of the east Qilian Mountains (Region C in S3 Fig), but during 1971–1990, only two strains were isolated in Region B (S3 Fig), and the subgroup appears to have shifted to Huangnan region (Region D in S3 Fig). During the 1990s, only three 1.IN2B strains were isolated in Region D (S3 Fig), and after 2000, no 1.IN2B strains were isolated across the whole of Qinghai Plateau (Fig 4A), suggesting that this subgroup of *Y*. *pestis* has become dormant or even extinct. Strains of 1.IN2C were initially isolated to the east of Qinghai Lake, and the regions closely surrounding the Qinghai Lake (1954–1970), and then spread to the west of Qinghai Lake during 1971–1990. From 1990 onwards, 1.IN2C strains appeared to have left the area surrounding Qinghai Lake and spread to regions to the west and south of it.

## Discussion

We have shown that there is a wide geographic distribution of *Y*. *pestis* in Qinghai Plateau. It is distributed mainly in the northeast, mid-east and southwest regions, including the southern foot of the Qilian Mountains, surrounding the Qinghai Lake region, the eastern Qaidam Basin, the Huangnan region, and Yushu Plateau (Fig 1B and S3 Fig). This distribution of *Y*. *pestis* is related to the distribution of its primary reservoir, *M*. *himalayana*. It is speculated that *M*. *himalayana* played an important role in the evolution of *Y*. *pestis* from *Y*. *pseudotuberculosis* [23]. As expected, no *Y*. *pestis* strain was isolated from the Hoh Xil, the biggest nature reserve in China, situated in the west of Qinghai Plateau, because of its high altitude and low population density of *M*. *himalayana*. Interestingly, although a high *M*. *himalayana* population density has been observed in the southeast part of Qinghai Plateau, epidemiological surveillance failed to obtain any isolates of *Y*. *pestis* from the reservoirs in this region; only positive serologies of *Y*. *pestis* F1 antibody have been detected [24]. Further investigation is required to confirm the presence of a natural plague focus in this region.

Qinghai Plateau played a critical role in ancient commercial exchange in China because it was the intersection between the main ancient trade routes, including the Silk Road, the Tang-Tibet Ancient Road, and the Tea Horse Road (Delamu). Therefore, the coexistence of multiple phylogenetic groups of *Y*. *pestis* in Qinghai Plateau might be related to human activities, such as cultural and commercial exchange between Qinghai and Tibet, which transferred plague pathogens between regions [10]. However, the long-distance spread of *Y*. *pestis* by human activity appeared to have a less important role in shaping plague foci during the modern age. The occasional human-related long-distance *Y*. *pestis* transmission event observed in this study, such as 1.IN2C_13 (Fig 3C), did not expand the original plague focus or establish a new population after transmission. In the current era, the influence of human activities on the distribution of plague foci seems to act in a different way, by driving transmission of *Y*. *pestis* through changing its local niche and destroying the natural environment that is necessary for *Y*. *pestis* survival. Before the 1970s, plague outbreaks were reported mainly in the eastern part of Qinghai Plateau, but subsequently, frequency of epidemics in the region gradually reduced and their occurrence began to shift towards the west of Qinghai Lake. After the 1990s, no *Y*. *pestis* strains could be isolated from the east side of Qinghai Plateau and the region closely encircling Qinghai Lake, which is the current economic center of Qinghai Province and the location of its capital city (Fig 4). Possible reasons for the extinction of *Y*. *pestis* in this region include active anti-plague interventions that have been implemented there following each outbreak; agricultural development, such as rapeseed cultivation, and development of tourism in the Qinghai Lake region, such as the international Tour of Qinghai Lake cycling race, held since 2002. All these activities reduce marmot population density, which in turn impacts the natural focus of *Y*. *pestis*, driving it towards the west of Qinghai Plateau, where there is much lower human population density, compared with the east.

Understanding the diversity of *Y*. *pestis* and its phylogeographic distributions will help in the design of tailored interventions for plague control. In Qinghai Plateau, we found seven *Y*. *pestis* groups that coincided with those we previously reported based on SNP analysis [10]. Group 1.IN2 is the dominant genotype in Qinghai Plateau, with a few minor groups scattered among the dominant ones. The non-dominant genotypes are genetically distinct from the dominant ones surrounding them, indicating that the non-dominant genotypes have possibly been transmitted from other regions, rather than having descended from the nearby dominant genotype. Our finding that one dominant group and several non-dominant groups coexist in Qinghai Plateau is consistent with a previous observation that major and minor genomovars of *Y*. *pestis* coexist in the majority of natural plague foci in China [17]. The dominant groups should play a greater role in sustaining a plague focus, while the non-dominant groups are sporadic and play a lesser role in maintaining its stability.

MLVA and CRISPR methods provide higher resolution than 25 selected SNPs in distinguishing the dominant *Y*. *pestis* population. The MLVA method splits the 1.IN2 group into three subgroups, interestingly, we observed a geographically clustered distribution of different *Y*. *pestis* subgroups in Qinghai Plateau. Host adaptation is one of the potential drivers to shape distribution of *Y*. *pestis*, however, in all 48 animal-derived 1.IN2 strains, 79.17% are isolated from *M*. *himalayana* (Fig 3). The remaining 10 strains were from five different species of mammals and distributed throughout the phylogenetic tree, suggesting no obvious evidence for host adaptation-derived evolution for *Y*. *pestis* in this region. The geographically constrained bacteriophage might be another possible driver that has led to the current distribution of 1.IN2 subgroups. It is known that the spacers in CRISPR loci are the legacy of the battle between a bacterium and bacteriophages [25]. Each geographically-specific 1.IN2 subgroup could be determined by specific spacers, suggesting the bacteriophage that carried the spacer-identical sequences is present in the corresponding regions. To explore the role of phages in microevolution of *Y*. *pestis* further, large scale sampling and sequencing of bacteriophage is needed in future work.

In addition to these findings, we observed periodic fluctuations in epidemics caused by different *Y*. *pestis* subgroups, such as 1.IN2A and 1.IN2C, suggesting that possible periodic variations in the size of each subpopulation might be influenced by many factors, including climate, vegetation, reservoir populations, vector distributions and bacteriophages. This emphasizes that future plague surveillance should collect a wider range of data than currently, so that improved and refined plague prevention and control measures can be designed and implemented.

In summary, the plague-endemic region of Qinghai Plateau still has considerable risk of outbreaks, especially in central and western areas, threatening transmission to other regions of China and worldwide. Systematically understanding the phylogeographical features of *Y*. *pestis* in this region will help us to implement countermeasures to prevent and control this deadly disease.

## Supporting information

S1 Table *Tsp*-dependent PCR primers used. (XLSX)

S2 Table Conventional PCR primers for verifying the status of SNPs. (XLSX)

S3 Table VNTR loci and primers. (XLSX)

S4 Table Genotypes of 102 *Y*. *pestis* isolates in Qinghai Plateau. (XLSX)

S1 Fig Schematic of the minimum spanning tree of *Y*. *pestis*. Circles represent the 23 SNP groups identified by Morelli and Cui *et al* [9, 10]. Black dots between two groups indicate 25 SNPs that were identified by PCR using GenoType *Tsp* DNA Polymerase. Underlining highlights the SNPs, which were further confirmed using conventional PCR and Sanger sequencing.
(TIF)

S2 Fig Procedure for identifying SNPs using GenoType *Tsp* DNA Polymerase. Two forward primers, wildtype forward primer (WF primer) and mutant forward primer (MF primer), which paired with the same common reverse primer (CR Primer), were designed. There are single nucleotide variations at the 3’-terminal between the WF primer and the MF primer. Two accompanying PCRs, using different primer combinations (WF primer paired CR primer and MF primer paired CR primer), were performed using *Tsp* DNA Polymerase, according to the instruction Platinum GenoTYPE *Tsp* DNA Polymerase. The products of PCR were subjected to electrophoresis using agarose gel. Amplification results display the SNP state of the test strains. If the SNP loci of the test stain match the 3’-terminal base of the forward primer, a positive band is displayed, otherwise it is a negative result.
(TIF)

S3 Fig Map of Qinghai Plateau. Black numbers denote the 32 counties or districts from where 102 isolates of *Y*. *pestis* were obtained. White numbers denote the 11 counties from which no *Y*. *pestis* strain had been isolated. It is notable that, despite no isolate being identified, serum positive for *Y*. *pestis* F1 antibody was detected from *M*. *himalayana* in the location marked 31. Colors highlight the four major regions where the majority of *Y*. *pestis* strains were isolated. A: Yushu Plateau, including Yushu, Nangqên, Zadoi (partial), Zhidoi (partial), Qumarleb (partial), and Chindoi county. B: Region surrounding Qinghai Lake plus the eastern Qaidam Basin, including Huangzhong, Huangyuan, Guide, Haiyan, Gangca, Gonghe, Xinghai, Wulan, Tianjun, and Delhi County. C: The southern foot of the east Qilian Mountains, including Qilian, Menyuan, and Datong County. D: Huangnan region, including Xunhua, Tongren, Zêkog, Maqên, Tongde, Guinan, and Henan County. The capital city, Xining, of Qinghai province, is marked with a red star.
(TIF)

S4 Fig Natural plague foci on or surrounding Qinghai Plateau. Six natural plague foci, on or surrounding Qinghai Plateau, are indicated in different colors on the map [17]. Focus C: *Marmota himalayana* plague focus of the Qinghai-Gansu-Tibet Grassland; Focus D: *Marmota himalayana* plague focus of the Qilian Mountains; Focus G: *Marmota himalayana* plague focus in the Gangdisi Mountains; Focus J: *Spermophilus dauricus alaschanicus* plague focus of the Loess Plateau in Gansu and Ningxia provinces; Focus K: *Marmota himalayana* plague focus of the Kunlun Mountains; Focus M: *Microtus fuscus* plague focus in Qinghai and Sichuan provinces. Gray fonts indicate different provinces in China.
(TIF)
