# Supplementary figures and images for: Genetic diversity and spatial-temporal distribution of Yersinia pestis in Qinghai Plateau, China
Source: PLoS Negl Trop Dis. 2018 Jun 25;12(6):e0006579. doi: 10.1371/journal.pntd.0006579 (PMC6034908; doi:10.1371/journal.pntd.0006579)

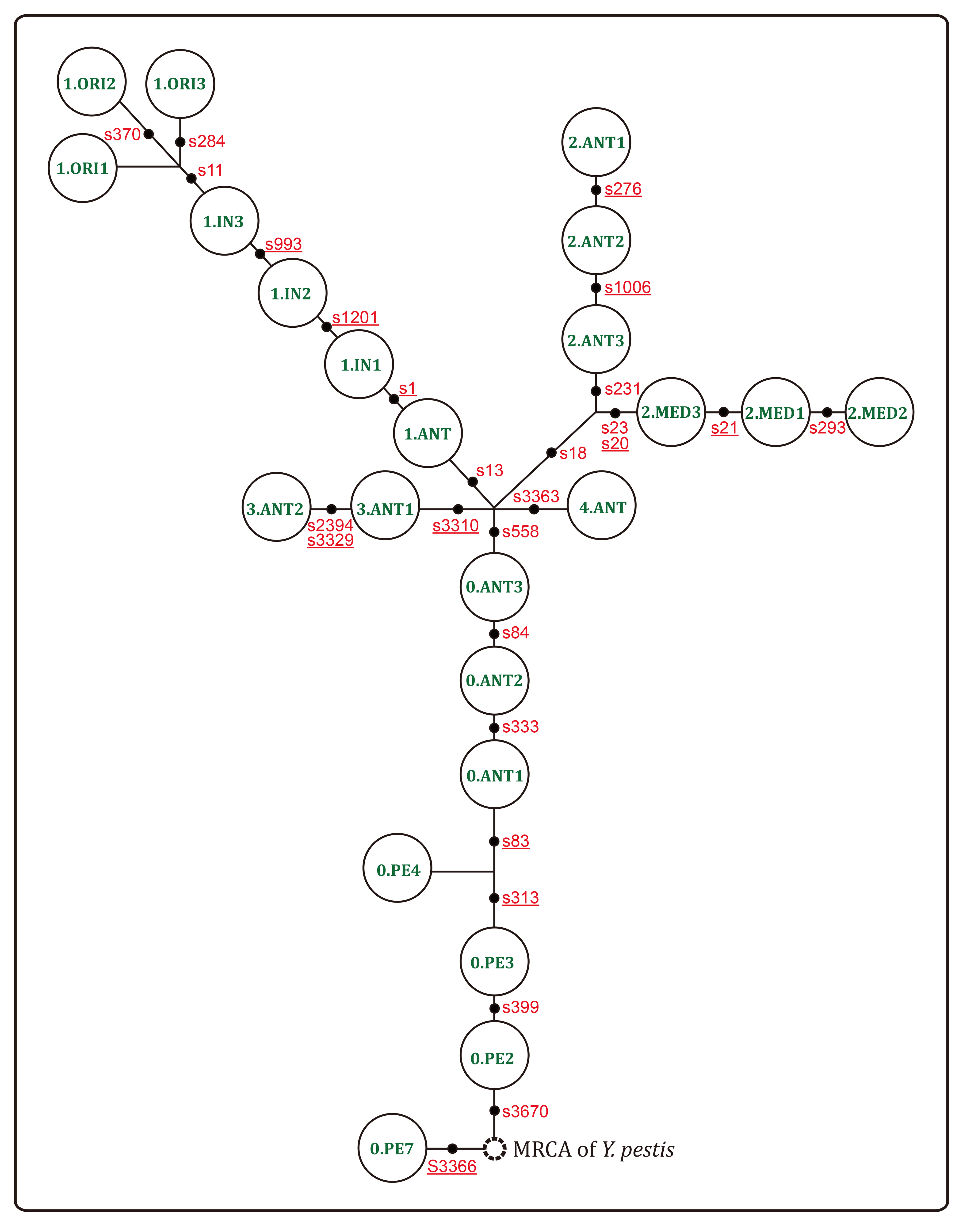

Supplement: S1 Fig — Circles represent the 23 SNP groups identified by Morelli and Cui et al [9, 10]. Black dots between two groups indicate 25 SNPs that were identified by PCR using GenoType Tsp DNA Polymerase. Underlining highlights the SNPs, which were further confirmed using conventional PCR and Sanger sequencing. (TIF) [file pntd.0006579.s005.tif]

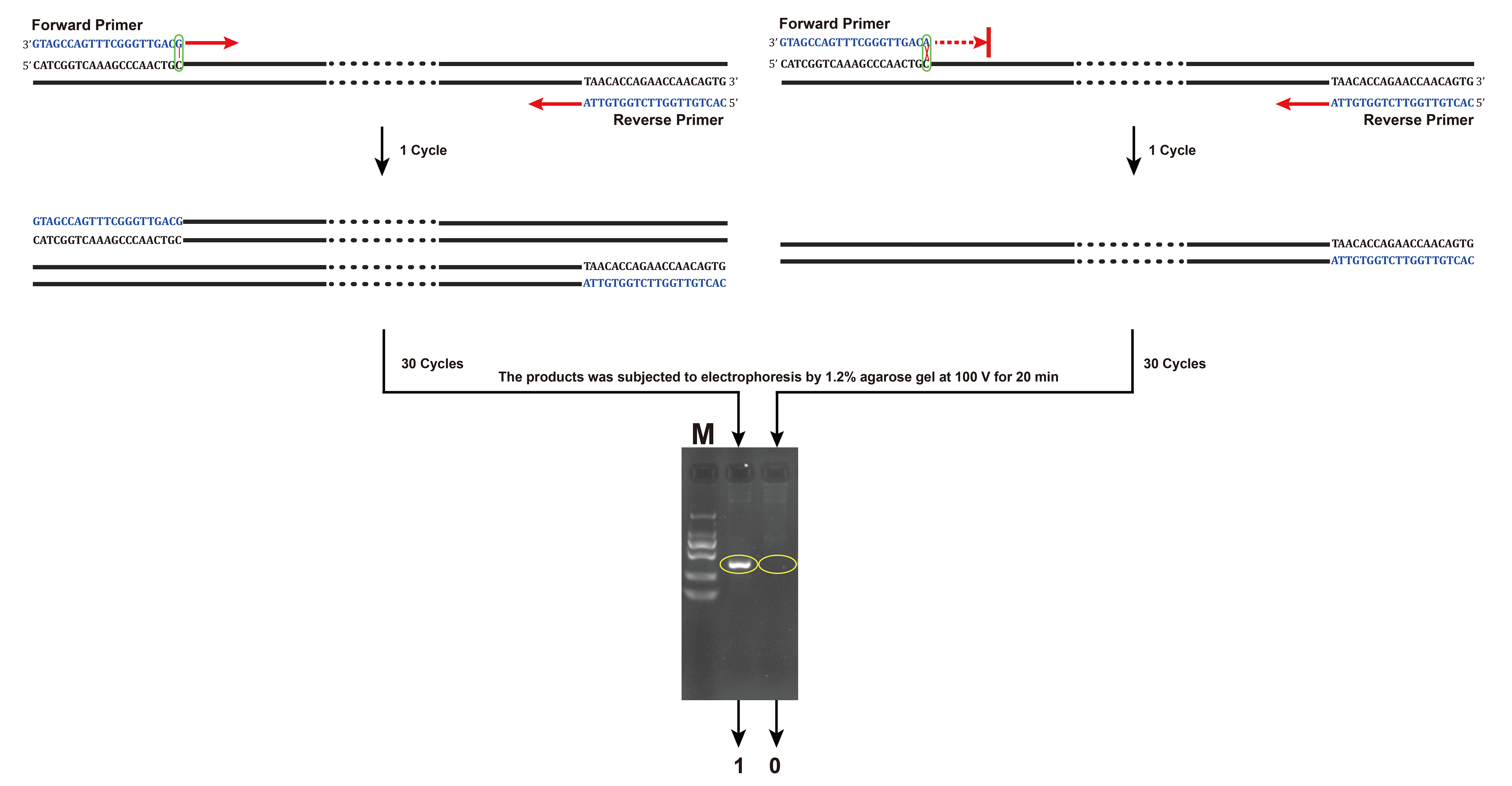

Supplement: S2 Fig — Two forward primers, wildtype forward primer (WF primer) and mutant forward primer (MF primer), which paired with the same common reverse primer (CR Primer), were designed. There are single nucleotide variations at the 3’-terminal between the WF primer and the MF primer. Two accompanying PCRs, using different primer combinations (WF primer paired CR primer and MF primer paired CR primer), were performed using Tsp DNA Polymerase, according to the instruction Platinum GenoTYPE Tsp DNA Polymerase. The products of PCR were subjected to electrophoresis using agarose gel. Amplification results display the SNP state of the test strains. If the SNP loci of the test stain match the 3’-terminal base of the forward primer, a positive band is displayed, otherwise it is a negative result. (TIF) [file pntd.0006579.s006.tif]

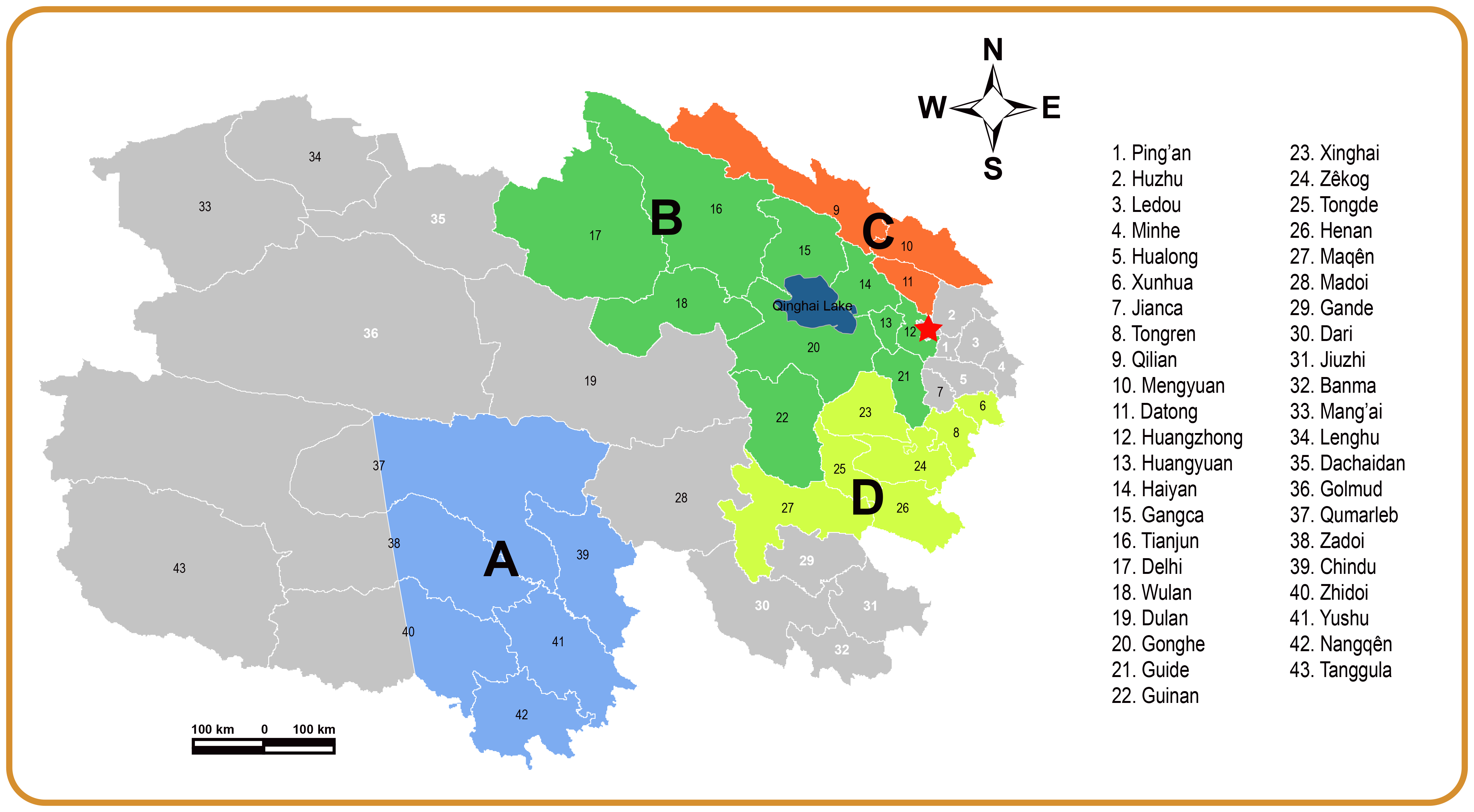

Supplement: S3 Fig — Black numbers denote the 32 counties or districts from where 102 isolates of Y. pestis were obtained. White numbers denote the 11 counties from which no Y. pestis strain had been isolated. It is notable that, despite no isolate being identified, serum positive for Y. pestis F1 antibody was detected from M. himalayana in the location marked 31. Colors highlight the four major regions where the majority of Y. pestis strains were isolated. A: Yushu Plateau, including Yushu, Nangqên, Zadoi (partial), Zhidoi (partial), Qumarleb (partial), and Chindoi county. B: Region surrounding Qinghai Lake plus the eastern Qaidam Basin, including Huangzhong, Huangyuan, Guide, Haiyan, Gangca, Gonghe, Xinghai, Wulan, Tianjun, and Delhi County. C: The southern foot of the east Qilian Mountains, including Qilian, Menyuan, and Datong County. D: Huangnan region, including Xunhua, Tongren, Zêkog, Maqên, Tongde, Guinan, and Henan County. The capital city, Xining, of Qinghai province, is marked with a red star. (TIF) [file pntd.0006579.s007.tif]

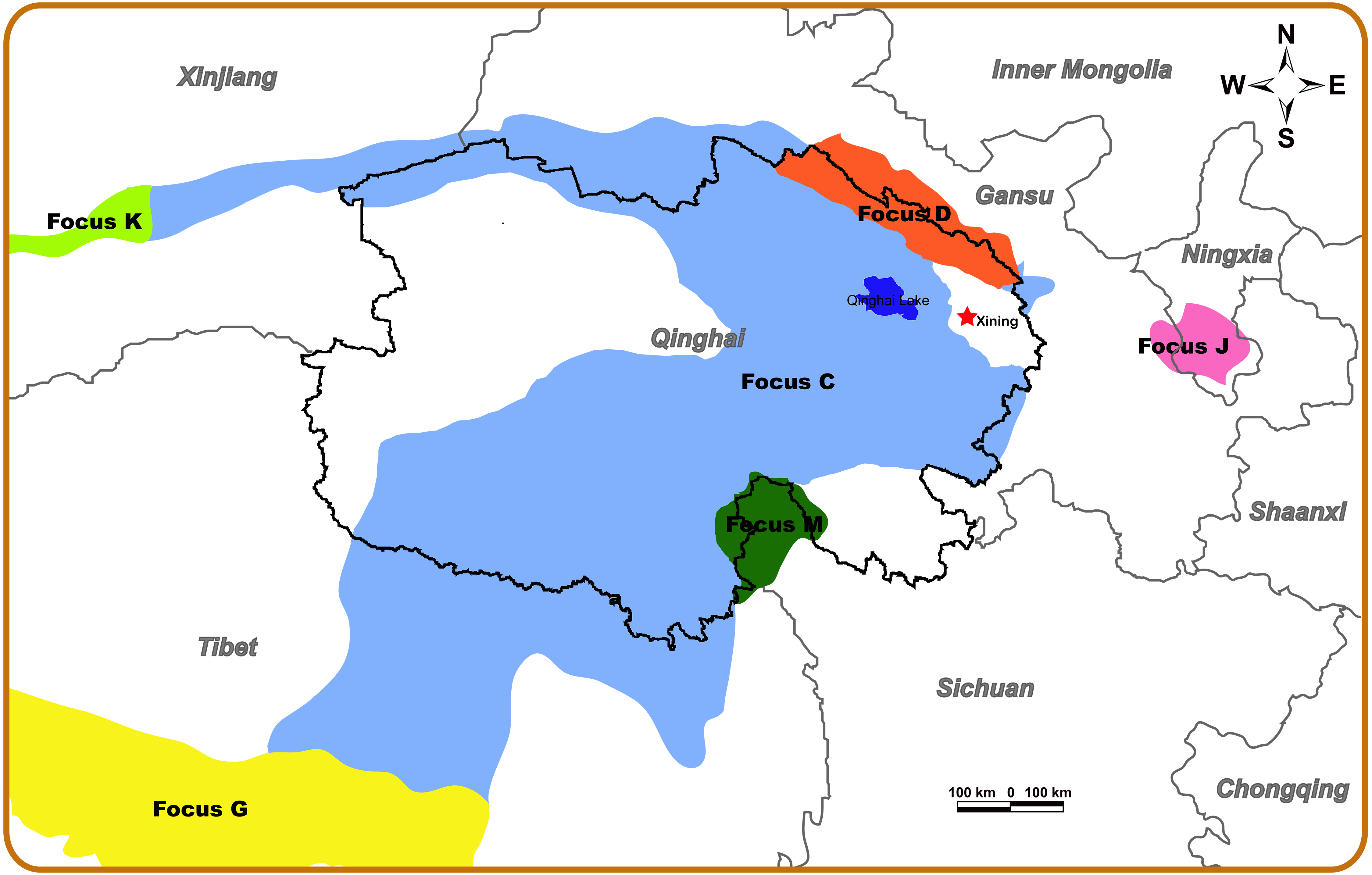

Supplement: S4 Fig — Six natural plague foci, on or surrounding Qinghai Plateau, are indicated in different colors on the map [17]. Focus C: Marmota himalayana plague focus of the Qinghai-Gansu-Tibet Grassland; Focus D: Marmota himalayana plague focus of the Qilian Mountains; Focus G: Marmota himalayana plague focus in the Gangdisi Mountains; Focus J: Spermophilus dauricus alaschanicus plague focus of the Loess Plateau in Gansu and Ningxia provinces; Focus K: Marmota himalayana plague focus of the Kunlun Mountains; Focus M: Microtus fuscus plague focus in Qinghai and Sichuan provinces. Gray fonts indicate different provinces in China. (TIF) [file pntd.0006579.s008.tif]
